# Knowledge of diagnosis and management of selected oral mucosal lesions among dentists in The Netherlands

**DOI:** 10.4317/medoral.25774

**Published:** 2023-01-15

**Authors:** Marcella R Poelman, Henk S Brand, Milad Asadi, Sharon Remmelzwaal, Derk H Jan Jager, Jan GAM de de Visscher

**Affiliations:** 1 Department of Oral and Maxillofacial Surgery/Oral Pathology, Amsterdam UMC location Vrije Universiteit/Academic Centre for Dentistry Amsterdam (ACTA), Amsterdam, The Netherlands; 2 Centre for Special Care Dentistry, Amsterdam, The Netherlands; 3 Department of Oral Biochemistry, Academic Centre for Dentistry Amsterdam (ACTA), Amsterdam, The Netherlands; 4 Department of Epidemiology and Biostatistics, Amsterdam UMC location Vrije Universiteit Amsterdam, Amsterdam, The Netherlands

## Abstract

**Background:**

Knowledge of oral mucosal lesions (OMLs) among dentists is relevant in diagnosing potentially malignant diseases and oral cancer at an early stage. The aim of this survey was to explore dentists’ knowledge about OMLs.

**Material and Methods:**

Respondents to a web-based questionnaire, containing 11 clinical vignettes representing patients with various OMLs, provided a (differential) diagnosis and management for each. Information about demographics and clinical experience of the participants was acquired as well. Descriptive statistics were performed and T-tests were used to test for significant (*p*<0.05) differences in mean scores for correct diagnosis and management between subgroups based on demographic variables.

**Results:**

Forty-four of 500 invited dentists completed the questionnaire. For (potentially) malignant OMLs, the number of correct diagnoses ranged from 14 to 93%, whilst the number of correct management decisions ranged from 43 to 86%. For benign OMLs, the number of correct diagnoses and management decisions ranged from 32 to 100% and 9 to 48%, respectively. For 11 clinical vignettes, mean scores for correct diagnosis, correct management and correct diagnosis and management were respectively 7.2 (±1.8), 5.7 (±1.5), and 3.8 (±1.7).

**Conclusions:**

The results show that dentists in the Netherlands do not have sufficient knowledge to accurately diagnose some OMLs and to select a correct management. This may result in over-referral of benign OMLs and under-referral for (potentially) malignant OMLs. Clinical guidelines, that include standardized criteria for referral, and continuing education, may improve dentists’ ability to correctly diagnose and accurately manage OMLs.

** Key words:**Oral mucosal lesion, dentists’ knowledge, mouth diseases, oral potential malignant disease, referral and consultation.

## Introduction

Knowledge of oral mucosal lesions (OMLs) among dental practitioners is relevant in diagnosing relatively harmless lesions but also oral cancer at an early stage and their potentially malignant precursors. In case of malignant disease, early detection reduces mortality and morbidity (1). OMLs prevalence, defined as an abnormal change in the oral mucosa such as the colour of surface, swelling or loss of integrity (2), varies significantly across different studies, ranging from 5% to 65% (3). The variability arises from differences in methodology employed in studies and from sociodemographic differences between countries (4).

OMLs include developmental defects, benign lesions, oral potential malignant disease (OPMD) and malignant disease. These various diseases may cause symptoms such as a burning sensation, swelling, irritation or pain, but often do not cause any symptoms (2,5-7). Especially in oral squamous cell carcinoma (OSCC), accounting for over 90% of oral cancers, symptoms are limited or lacking at an early stage of the disease, often causing patients to seek late care with cancer at an advanced stage (5,7,8). Screening patients for oral cancer during dental check-up appointments using visual oral examination is a cost-effective strategy in oral cancer detection (9). Since dental practitioners see many patients on a regular basis, they have the opportunity to detect OPMD and early stage OSCC, and to determine which lesions with a provisional diagnosis can be closely observed versus referral as cancer is suspected. A recent systematic review about delay in diagnosis of oral cancer indicated that lack of ability to correctly diagnose OMLs among healthcare professionals is related to delay in diagnosis (10). Various studies explored oral cancer knowledge, attitudes and screening practices of dental practitioners, with only a few focusing on referral decisions (11-14). One approach that has proved very effective in teaching examining diagnostic and referral decisions has been the use of clinical vignettes (15,16). These simulate clinical situations by describing a patients’ visit by using clinical pictures, a description of the complaint, and a patients’ history. Using these, it was found that the number of correct referral decisions by dental practitioners in England was higher than the number of accurate diagnoses There also appeared to be a lack of discrimination between risk factors in the process of making a referral decision (16). It was suggested that when dentists are in doubt about the diagnosis of OMLs, their default position is to refer. The latter and other studies on this topic, mainly focussed on OPMDs and OSCCs whereas some benign OMLs may have the potential to negatively influence the quality of life through impact on mastication, swallowing, aesthetics, and speech (5,12,16,17). Some OPMDs or OSCC at an early stage mimic benign OMLs, such as small tumours or ulcerative lesions that are diagnosed as traumata, which causes a delay in diagnosis (10). So, ability to distinguish between these types of OMLs is important.

To explore dentists’ knowledge about OMLs, we conducted this survey among dentists in the Netherlands, using a questionnaire containing 11 clinical vignettes, which represented various OMLs and required respondents to provide a (differential) diagnosis and accurate management for each.

## Material and Methods

- Data collection

By means of its Data Stations Project, the Royal Dutch Dental Association (KNMT) periodically collects data on delivery of oral health care, practice management and dentists’ opinions and views regarding current issues in dentistry in the Netherlands (18). In January 2019, an invitation e-mail was sent to 500 dentists (243 males; 257 females), randomly selected from the dentists who participate in the Data Stations Project periodic surveys. The e-mail included a link to the 25-item web questionnaire. Reminders were sent after 2 and 4 weeks; data collection ended 6 weeks after the first invitation.

- Instrument

**Figure 1:**
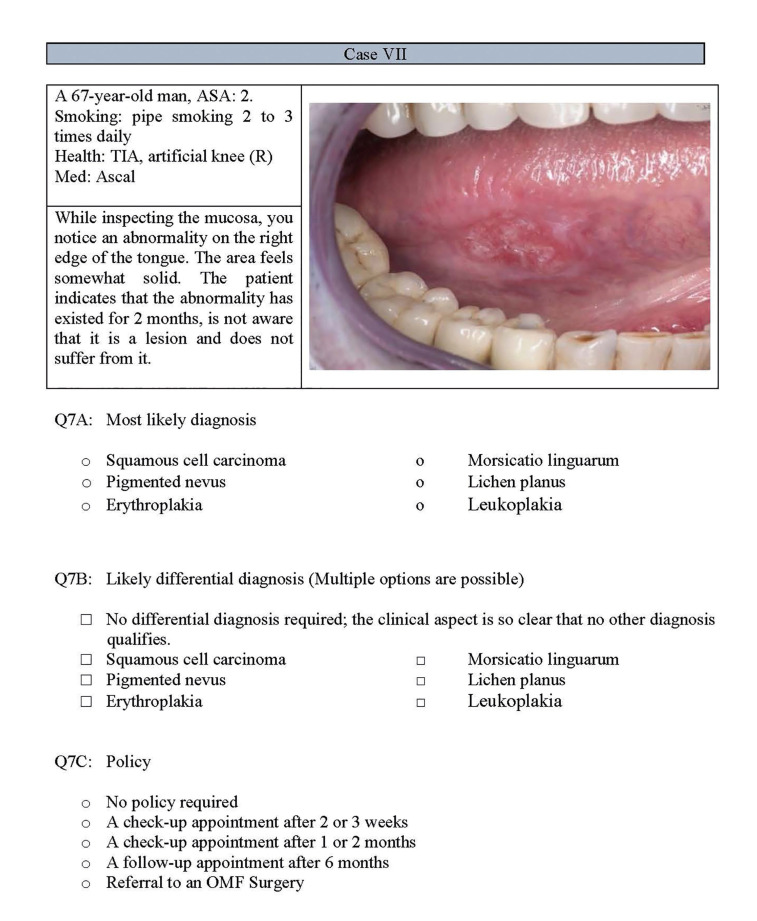
Example of a vignette used in the questionnaire.

The 25-item self-constructed questionnaire (Supplement 1) in Dutch consisted of two sections and was developed for this study in cooperation with the department of Oral and Maxillofacial Surgery of the Amsterdam University Medical Centre in Amsterdam. The first section consisted of 11 clinical vignettes, representing patients with various OMLs including a clinical picture of a lesion and a brief textual description of a simulated clinical history. The description contained typical characteristics for each OML (for an example of a clinical vignette, see Fig. 1). These pictures and descriptions were provided by a maxillofacial surgeon and selected from medical records of referrals to the outpatient clinic. The selected cases represented a wide range of benign and (potentially) malignant OMLs; white lesions [2] red lesions [2], pigmentations [2], ulcerated lesions [2], soft tissue enlargements [2] and one skin lesion (Tab. 2). In the first section of the questionnaire three multiple choice questions for each case required participants to select the correct diagnosis, differential diagnosis, and management. While for the diagnosis and management one option could be selected, for the differential diagnosis, multiple options could be selected. The multiple-choice questions were constructed using information the guidelines of the Dutch Association of Oral and Maxillofacial Surgeons. The second section contained 7 questions and included demographic characteristics, participants’ current oral examination practices, referral practices, and post-graduate education courses.

A panel of fifteen dental students, eight residents of the department of Oral and Maxillofacial Surgery and three maxillofacial surgeons of the department of Oral and Maxillofacial Surgery, pilot tested the questionnaire and minor revisions were made upon their feedback.

- Statistical analysis

Data were analysed using Statistical Package for Social Sciences (SPSS), version 26 (IBM Inc., New York, USA). Figures were made using GraphPad Prism version 9.3.1 for Windows, GraphPad Software, San Diego, California USA, www.graphpad.com. Frequencies and means (SD) were used for data description. To grade knowledge, participants were awarded one point for each correct answer: correct diagnosis (CD), correct management (CM) and the combination of correct diagnosis and management (CD+M). To test whether there were significant (*p*<0.05) differences in mean scores of CD, CM and CD+M between subgroups based on the variables sex, years of graduation, and participants that did and did not want to attend a course on various aspects of OMLs, unpaired t-tests were used.

- Ethics statement

The invitation e-mail mentioned that participation was voluntary. Participants consented to the survey by answering the questionnaire, which was distributed by an independent data collection institute that was responsible for confidential processing of the data. Data were anonymised before they were sent to the researchers. The Medical Ethical Review Committee of the VU University confirmed that the Medical Research Involving Human Subjects Act did not apply to this study, so an institutional review board approval was not necessary.

## Results

A number of 500 questionnaires was distributed among dentists, and completed by 63 participants. After screening for incomplete data, 19 were removed and data of the remaining 44 participants were used for analysis, resulting in a response rate of 8.8%. The complete demographic dataset is summarised in Table 1.

In the benign group, vignette 8 (aphthous stomatitis) had the highest percentage (100%) of correct diagnosis, whilst the lowest (32%) was case 6 (melanotic macule). For vignette 9 (fibroma), the correct management, a follow-up appointment after 6 months, was selected by 48% of participants. Only 9% of participants correctly reported that there was no follow-up up appointment necessary for vignettes 3 (amalgam tattoo) and 5 (median rhomboid glossitis (MRG)), respectively) (Table 2).

In the (potentially) malignant group, the most frequently correct diagnosed was vignette 2 (oral lichen planus; 93% correct), whilst vignette 11 (cutaneous SCC), was diagnosed correctly by 14%. Except for oral lichen planus, all (potentially) malignant OMLs required referral, which was selected for 73-86% of the vignettes (Table 2).

For vignettes that represented patients who should be referred to specialist care, the correct management varied from 59 to 86%. Almost all dentists would directly refer the patients in vignette 7 and 11, which represented OSCC and cutaneous SCC (both 86%), or make a check-up appointment after 2-3 weeks (11% and 14%). For vignettes that did not need referral, the most commonly selected incorrect management was direct referral to specialist care; except for aphthous stomatitis (Table 2).

**Table 1:**
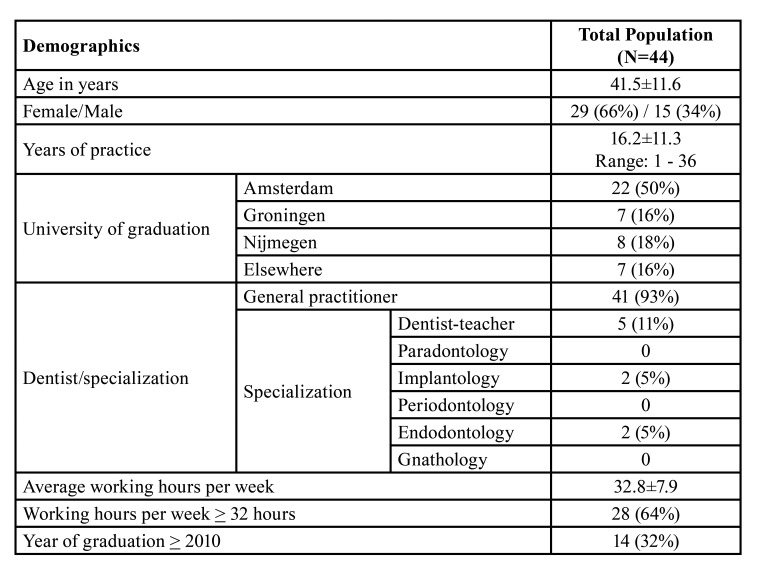
Demographics of the study population. Continuous variables are depicted as mean±SD and categorical variables as N(%).

**Table 2:**
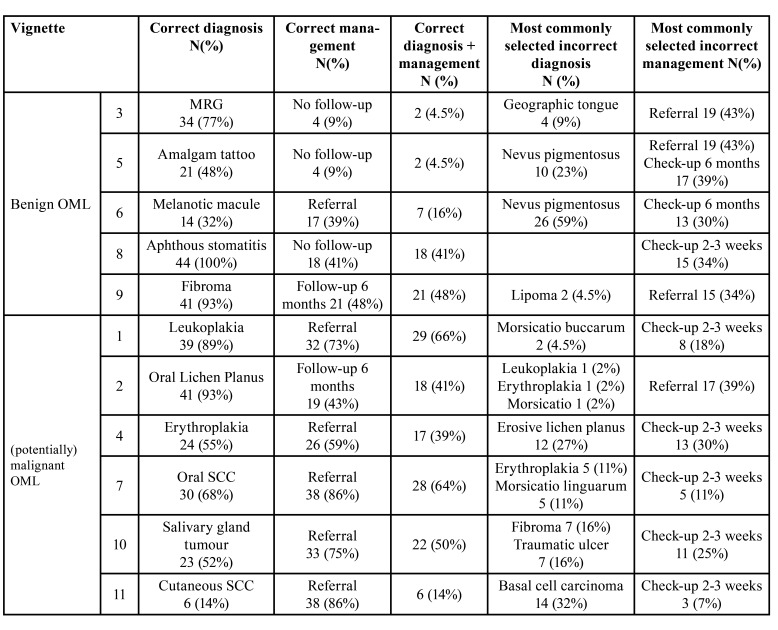
Numbers and percentages of participants that selected the correct diagnosis, follow-up management and diagnosis+management and the most commonly selected misdiagnosis and incorrect management, for 11 clinical vignettes.

**Figure 2:**
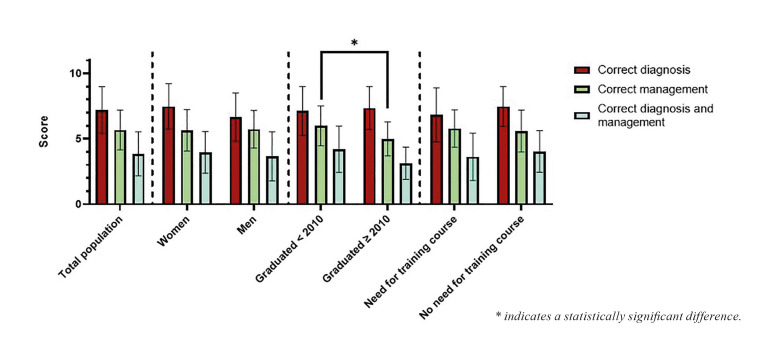
Mean number of correct diagnosis, correct management and correct diagnosis and management, stratified for sex, year of graduation and expressed need of education (data expressed as mean±SD).

Mean scores for correct diagnosis (CD), correct management (CM) and correct diagnosis and management (CM+P) were respectively; 7.2 (±1.8), 5.7 (±1.5) and 3.8 (±1.7) of 11 vignettes. This means that on average participants identified the CD for 7.2 of 11 vignettes, the CM for 5.7 vignettes and the CD+M for 3.8 vignettes. For the mean score for CM, a significant difference was found between dentists that graduated after 2010 and before 2010; the latter more often selected the correct management (t(42) = -2.11, *p*=0.04). There were no statistically significant relationships between the CD, CM and CD+M scores and sex, and between dentists that did and did not express a need for continuing education (Fig. 2).

Of the participants, 65.9% reported inspecting the oral mucosa in every patient visiting for a check-up appointment, 27.3% in most patients and 6.8% in some patients. Sixty-three percent reported taking a clinical photograph when observing an OML in their patients. Most participants (72.7%) mentioned that to help them making a diagnosis they sometimes use clinical images from various sources especially the internet (38.6%) and textbooks (40.9%). Two thirds (63.6%) of participants referred one to five patients a year to specialist care and 20.5% referred six to ten patients per year. The main reason for referring a patient was confirmation of a diagnosis (90.9%).

At the moment of the survey, 84.1% of respondents expressed no need for continuing education about OMLs, and 77% completed a post-graduate education about this topic in the past (Table 3).

**Table 3:**
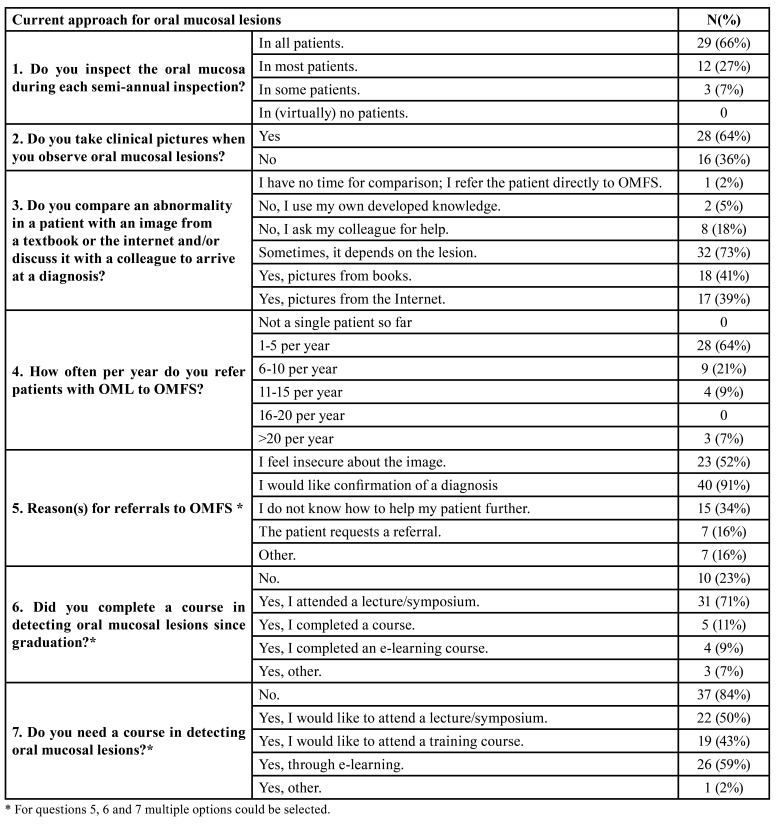
Current approach for oral mucosal lesions.

## Discussion

The aim of this survey was to explore whether dentists’ knowledge about OMLs is sufficient to correctly diagnose and manage various OMLs in patients. Therefore, we conducted this survey among dentists in the Netherlands, using a questionnaire containing 11 clinical vignettes, which represented selected OMLs and required respondents to provide a (differential) diagnosis and accurate management for each. In summary, the results revealed a lack of ability to correctly diagnose some OMLs and a tendency to refer to a specialist when in doubt. This tendency was evident from the fact that there were more correct referrals for malignant OMLs than correct diagnoses. For three vignettes representing malignant OMLs, 14 to 68% of participants selected a correct diagnosis, whereas 75 to 86% selected the correct management of direct referral. So, although participants lacked knowledge to provide a correct diagnosis, they selected the correct management. For benign OMLs, the opposite was observed; regardless of a correct diagnosis, many respondents reported referring a patient with a benign OMLs to a specialist. Remarkably, this tendency was not present for the vignette representing oral lichen planus, which we categorised as a potentially malignant lesion, which is arguable, as the overall malignant transformation rate is low and varies from 0.5-1% (19). Many respondents probably had this knowledge since the majority selected the correct management of a check-up appointment after 6 months, instead of direct referral. In addition, respondents reported that the main reason for referral to a specialist was confirmation of their diagnosis (91%), which suggests that they do not feel confident about their diagnosis.

The lack of ability in diagnosing some OMLs was evident as the range of correct diagnoses was 14 to 100% and the fact that on average only 7.2 out of 11 cases were diagnosed correctly. The most common benign OMLs, aphthous stomatitis (20), was diagnosed correctly by all participants. The lowest percentage for correct diagnosis (14%), was for the vignette representing a cutaneous squamous cell carcinoma. This vignette was included to increase awareness among dentists about including examination of the skin of patients as part of their routine extra oral examination. Cutaneous SCC is the second most common cancer with an increasing incidence and it frequently occurs on the head and neck skin (21).

While most benign OMLs can be diagnosed based on clinical features only, for (potentially) malignant OML a biopsy for histopathological assessment is required (11). In some countries dentists are encouraged to take such biopsies. However, this is not the case in The Netherlands, so timely referral of (potentially) malignant lesions is even more important than providing an initial correct diagnosis.

Comparing results for specific OML to previous research is difficult, as results of this study are vignette specific; each lesion is a separate entity with a unique combination of characteristics. Comparable research is scarce, as studies that investigated dentists’ ability in diagnosing OMLs used questionnaires that did not include patients’ history and clinical presentation (22-25). We found one comparable study among dentists in England and our study confirms their findings that dentists have a tendency to refer to a specialist when in doubt about their diagnosis (16). It was not possible to compare results for specific OMLs because they only reported whether the diagnosis was correct or not and did not show the actual diagnosis for the clinical vignettes.

Our study contributes to the aim of diagnosing early-stage oral cancer in patients in two ways. First, it creates awareness among Dutch dentists about their limited ability to correctly diagnose and refer OMLs. Second, specification of deficits in diagnostic ability and management of OMLs among dentists is likely to be beneficial for adapting curricula of dental schools, as there is a wide variety in theoretical content and examination techniques taught in European dental schools (26). Results may also provide a background for development of clinical guidelines to be used by healthcare professionals to diagnose and manage OMLs and improvement of post-graduate education. Most respondents reported that they already had attended a post-graduate course about OMLs and did not need additional education. Considering the relatively low percentages of correct diagnosis and patient management, attending these courses more frequently might be beneficial to refresh knowledge about OMLs. Further research into the most appropriate content and forms of education, and frequency of post-graduate education is desirable. This study showed a tendency of over-referral, so evaluation whether providing dentists with clinical guidelines, that include standardized criteria for referral, could be beneficial. Research in the United Kingdom has showed that availability of a national referral guideline improves the cancer detection rate and reduces over-referral. However, compliance with guidelines is required and needs to be improved (27). Availability of an electronic consultation tool for dentists, with the possibility to consult an oral surgeon, is also an option (22,23).

Although the questionnaire in our study contained a limited number of 11 OMLs, to limit the time investment for participants, it still provides information about a wide range of OMLs that dentists are faced with in their daily practice.

For selection of a correct management for each vignette, we consulted existing guidelines of the Dutch Association of Oral and Maxillofacial Surgeons. Management and referral guidelines are sometimes not unanimous and may be interpreted differently. For example, for the vignette representing a salivary gland tumour, we decided that direct referral was the correct management. This is arguable because the textual description revealed that the lesion was present for only 1.5 weeks in this patient with complete dentures, so an alternative policy could be removal of a possible traumatic cause and making a follow-up appointment after 2-3 weeks. When considering this second option also correct, all participants selected a correct management for this vignette. A similar discussion regarding selection of the correct management applies to the vignettes representing leukoplakia and erythroplakia; the provided patients’ history did not mention how long these lesions were present. So, if we considered removal of possible traumatic causes, followed by an appointment after 2 to 3 weeks also correct, ability to select a correct management for these vignettes was excellent. Therefore, the mean scores for correct management for several OMLs should be interpreted with some caution. In addition, bias may have occurred in this survey, as participation was voluntarily, and dentists with an interest in this topic may have been more likely to participate, results may present a more optimistic picture. Only one statistically significant relationship was found for mean scores for CD, CM and CD+M between subgroups based on demographic characteristics, this may be due to the small sample size and low response rate. A postal survey may increase the response rate (28).

## Conclusions

In conclusion, this study shows that dentists in the Netherlands do not have sufficient knowledge of some OMLs in order to provide an accurate diagnose and management. On average, only 7.2 out of 11 clinical vignettes representing OML were diagnosed correctly. The percentage of correct diagnosis for (potentially) malignant OML ranged from 14 to 68%, and for benign OML from 32 to 100%. Participants tended to refer when in doubt about the diagnosis. Further research should focus on exploring whether providing dentists with clinical guidelines that include standardized criteria for referral and continuing education could improve their ability to correctly diagnose OMLs and accurately manage these lesions.
